# Azole potentiation in *Candida* species

**DOI:** 10.1371/journal.ppat.1011583

**Published:** 2023-08-31

**Authors:** Jan S. Stenkiewicz-Witeska, Iuliana V. Ene

**Affiliations:** Institut Pasteur, Université Paris Cité, Fungal Heterogeneity Group, Paris, France; Vallabhbhai Patel Chest Institute, INDIA

## Abstract

Fungal infections are rising, with over 1.5 billion cases and more than 1 million deaths recorded each year. Among these, *Candida* infections are frequent in at-risk populations and the rapid development of drug resistance and tolerance contributes to their clinical persistence. Few antifungal drugs are available, and their efficacy is declining due to the environmental overuse and the expansion of multidrug-resistant species. One way to prolong their utility is by applying them in combination therapy. Here, we highlight recently described azole potentiators belonging to different categories: natural, repurposed, or novel compounds. We showcase examples of molecules and discuss their identified or proposed mode of action. We also emphasise the challenges in azole potentiator development, compounded by the lack of animal testing, the overreliance on *Candida albicans* and *Candida auris*, as well as the limited understanding of compound efficacy.

## Introduction

**Fig 1:**
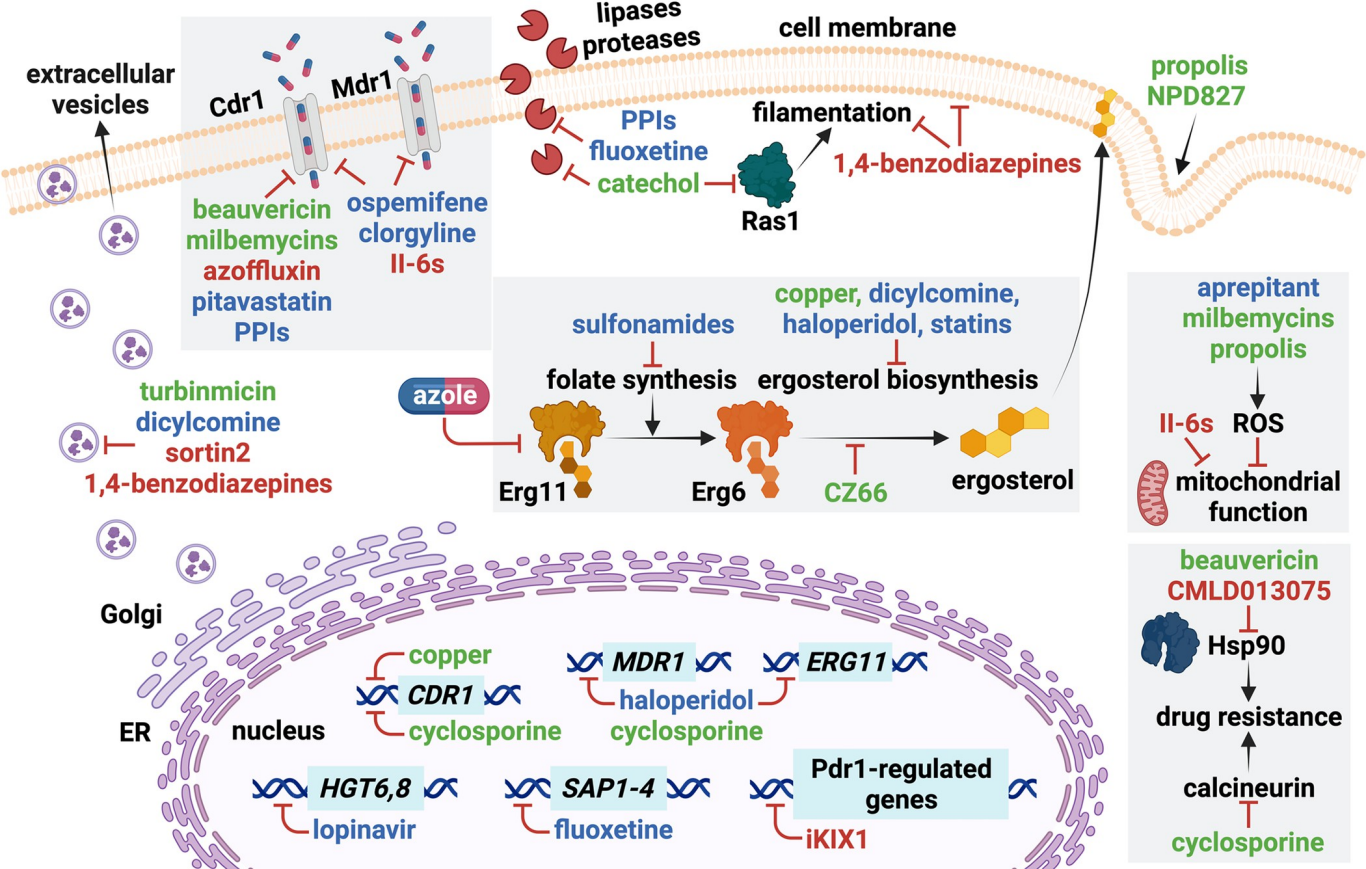
Overview of mechanisms of azole potentiation for compounds described in this review. The potentiator names are coloured according to the category they belong to—natural products (green), repurposed drugs (blue), or novel compounds (red). ER, endoplasmic reticulum. Blunt arrows (red) indicate inhibitory or disruptive effects. The image was created with BioRender.

Last year, the World Health Organization published its first fungal priority pathogen list, including *Candida albicans* and *Candida auris* in the critical importance group, and labelling other *Candida* species as either highly or moderately important [1]. Although certain *Candida* species are common human residents, they can cause fatal infections when the host immunity is impaired. Even more concerning is the widespread antifungal resistance within this genus, especially among *C*. *auris* isolates, which are often multidrug-resistant [2]. Azoles are the most widely used antifungals, due to their accessibility, low toxicity, and broad spectrum of action. They inhibit the ergosterol-synthesis enzyme encoded by *ERG11*, leading to the accumulation of toxic sterols, loss of membrane integrity, and growth arrest [3]. Prolonged azole treatment selects for drug resistance, often achieved via mutations of the target gene, drug transporters, or their respective regulators [3,4]. One strategy to extend the lifespan of antimicrobials is to use potentiators, which can enhance the activity of existing drugs and reduce the rate at which microbes gain resistance [5]. For fungi, they can also synergise with static drugs to render them cidal [6]. The pharmaceutical industry has been largely disinterested in the development of novel antifungals or their potentiators, likely due to the extreme development costs, as well as the slow and limited sales of compounds [4]. Thus, the weight of drug discovery has shifted towards the academic community. Here, we highlight molecules that potentiate azoles identified from natural, repurposed, or novel compounds (Fig 1). We discuss their mode of action, spectrum of activity, and identify the knowledge gaps that need to be addressed to advance their development.

## Natural products

Natural compounds have been used as therapeutics for most of human history and remain an important source of drug discovery. As pathogenic fungi target other animals, plants, or bacteria, these organisms have developed intrinsic defences that could be leveraged as antifungals. A key antifungal, amphotericin B, was first isolated from the bacterium *Streptomyces nodosus* in 1955 [7]. Similarly, beauvericin was isolated from the fungus *Beauveria bassiana* in 1969 and was shown to be active against bacterial, fungal, and animal cells [8]. Its efficacy as a potentiator was identified by screening natural product libraries with ketoconazole against *Candida parapsilosis* [9]. A combination of beauvericin and ketoconazole improved the survival of mice infected with *C*. *parapsilosis* [9]. In *C*. *albicans*, beauvericin inhibited drug efflux, but it also activated the protein kinase CK2 and inhibited the TOR1 complex and the global chaperone Hsp90 [10], thereby affecting azole resistance [11]. Despite the well-understood mechanism of action, the potential toxicity of beauvericin to human cells is a major challenge for its development as an azole potentiator.

Cyclosporine, a natural fungal product, is an approved immunosuppressant that acts by inhibiting calcineurin, a pathway essential for survival during membrane stress [12,13]. Furthermore, cyclosporine significantly decreased the expression of several azole resistance genes (including *ERG11*, *CDR1*, and *MDR1*) and increased intracellular calcium concentration [14]. Its combination with azoles was fungicidal and disturbed *C*. *albicans* biofilms [15,16]. However, the mammalian immunosuppressive activity of cyclosporine could render it harmful in combination therapies.

Milbemycins are metabolites produced by *Streptomyces* species, which are used as antiparasitics in veterinary medicine [17]. Milbemycin derivatives showed antifungal activity against *C*. *albicans* and *Nakaseomyces glabrata* (former *Candida glabrata*); they work by inhibiting ABC-mediated efflux and may promote cell death by increasing reactive oxygen species (ROS) [18]. Interestingly, milbemycin oxides were intrinsically fungicidal in vitro, effect which was enhanced in combination with azoles [18]. While milbemycin oxides were not effective on their own in vivo, the combination with azoles reduced fungal burdens in a murine infection model [18].

Turbinmicin has been identified through screening libraries of sea-dwelling microbial metabolites. Isolated from the microbiome of a sea squirt, turbinmicin inhibited the growth of several *Candida* species and decreased *C*. *auris* burdens in mice with disseminated infection [19]. Screening of a *Saccharomyces cerevisiae* mutant collection revealed Sec14, a protein involved in vesicular trafficking, as the target of turbinmicin [19]. Because it reduced extracellular vesicle production, turbinmicin impaired the extracellular matrix structure and the formation of *C*. *albicans* biofilms [20]. Similarly, screening of natural products identified a synergy between fluconazole and an imidazopyrazoindole, NPD827 [21]. This molecule displayed an unusual mode of action, increasing the mobility of the membrane and disrupting its integrity by associating with *C*. *albicans* sterols [21]. This resulted in vacuolar fragmentation and disruption of lipid recycling. The drug combination inhibited filamentation and blocked biofilm formation in a rat model of *C*. *albicans* catheter infection [21]. While turbinmicin and NPD827 both target biofilms, which are exceptionally difficult to treat, additional animal testing and pharmacokinetic/pharmacodynamic (PK/PD) studies are needed to establish their efficacy.

Propolis is used by honeybees in hive maintenance, suggesting it could have antimicrobial properties. Propolis ethanolic extracts inhibited the growth, filamentation, and biofilm formation of *Candida* species, whether used alone or in conjunction with azoles [22,23]. Cells treated with propolis, particularly when high in phenols, exhibited cell wall and membrane defects that resulted in cell death [23]. Flavonoids present in propolis could also exert antifungal effects by altering mitochondrial function and increasing the accumulation of ROS [24]. However, the composition of propolis is highly diverse, resulting in variable activity, therefore, isolating promising compounds and developing them into efficient potentiators could circumvent this issue.

Catechol is a plant benzenediol used in pesticide synthesis due to its antimicrobial properties [25]. This compound inhibited filamentation, blocked the initial adherence step in biofilm formation, and inhibited the proteolytic and lipolytic activities of *C*. *albicans* [26]. The decrease in filamentation was mediated by increased farnesol production and inhibition of the Ras1 signalling pathway [26]. Interestingly, catechol potentiated other azoles and polyenes, enhancing their fungistatic and fungicidal effects, respectively [26]. However, given the lack of infection outcomes studies, it is difficult to assess its in vivo potential.

Another example of plant-derived potentiators is CZ66, a synthetic derivative of berberine. CZ66 had no antifungal activity on its own but it was cidal to *C*. *albicans* in combination with fluconazole [27]. The compound synergized with diverse azoles across *Candida* species but not with caspofungin. The addition of exogenous ergosterol rescued the growth of CZ66 and fluconazole-treated cells, and the authors identified Erg251, an enzyme in the alternative pathway of ergosterol synthesis, as a potential target [27]. Thus, inhibition of both late and alternative pathways of sterol synthesis through combination therapy can destabilise the fungal membrane [27]. However, although CZ66 was not cytotoxic, it did not improve mouse survival during infection, indicating that further development of the molecule is needed.

Natural potentiators are not limited to organic molecules, but also include essential metals. Both sequestration and supplementation of copper potentiated fluconazole against *N. glabrata* and *C*. *albicans* [28,29]. Transcriptomic profiling revealed that even small fluctuations in copper were sufficient to alter *C*. *albicans* azole responses, as copper is crucial to an array of biological processes [30]. In *N*. *glabrata*, this combination decreased the expression of efflux pumps, inhibited ergosterol biosynthesis, and disrupted cell wall and membrane integrity [31]. Additional studies are needed to understand how copper potentiates azoles in *Candida* species and whether it could be used as a potentiator given its cytotoxicity at high concentrations [32].

Natural products hold vast promise for azole potentiation, but they face several challenges. For complex products such as propolis, the variability in chemical content can lead to altered efficacy. Meanwhile, toxins with antifungal activity could also have high toxicity in humans, due to similarities between eukaryotic cells. Therefore, understanding the mechanism of action and pharmacokinetic properties of these compounds is essential for developing them as safe antifungal potentiators.

## Repurposed drugs

The approach linked to the lowest risk involves repurposing existing drugs. This is primarily due to their well-known toxicity and PK/PD properties, which simplifies their introduction into clinical trials [33]. Additional testing and optimisation could be needed to bring the active concentration within the therapeutic range, which could be expensive and laborious. Nonetheless, accessing libraries of approved molecules is a viable method of discovering new potentiators.

Eldesouky and colleagues showcased this method and uncovered a synergy between the antibacterial sulfonamides and fluconazole. Sulphonamides inhibit an enzyme in the folate synthesis pathway and have been previously investigated as potential antifungals [34]. The study tested azole-resistant *C*. *albicans* isolates and found a synergy between sulfamethoxazole and fluconazole. This combination inhibited the growth of *C*. *albicans* and decreased fungal burdens in a *Caenorhabditis elegans* model of fungal infection [35]. Sulfonamides did not alter drug efflux, but they were antagonised by supplementation of para-aminobenzoic acid, a folic acid precursor [35]. *C*. *auris* cells lacking *ERG11* displayed increased sensitivity to this drug combination, but not efflux-activated strains, suggesting that the synergy was dependent on the mechanism of resistance [36]. Inhibition of folic acid biosynthesis could affect ergosterol synthesis through negative feedback on the Erg6 enzyme [37]. Thus, additional studies are necessary to define the mechanism of azole potentiation by sulfonamides.

Lopinavir, an HIV-1 protease inhibitor, synergized with itraconazole to reduce fungal burdens and increase survival of *C*. *elegans* during *C*. *auris* infection [38]. The drug combination down-regulated the expression of *HGT6* and *HGT8*, 2 high-affinity glucose transporters, and increased the expression of the lipid translocase *RTA3* [38]. Lopinavir interfered with glucose utilisation, lowered ATP levels, and reduced drug efflux in a dose-dependent manner [38]. As drug efflux is an energy-intensive process, lower ATP levels could translate to reduced efflux. Atazanavir and darunavir, 2 drugs from the same class, also showed antifungal activity against *C*. *albicans*, and inhibited filamentation and the expression of *SAP2* and *BCR1*, genes involved in virulence and biofilm formation, respectively [39]. In contrast to lopinavir, which did not increase host survival on its own, atazanavir and darunavir enhanced the survival of *C*. *albicans*-infected *Galleria mellonella* [38,39]. Combining atazanavir with itraconazole also restored azole susceptibility in *C*. *auris* [40]. Consequently, these anti-infectives could act differently in these *Candida* species, warranting the understanding of the mechanisms underlying their synergy with azoles.

Screening of the John Hopkins Clinical Compounds Library yielded aprepitant, an anti-emetic agent, as an azole potentiator [41]. In *C*. *auris*, the synergistic effect with itraconazole coincided with the down-regulation of glucose transporters but also of *FTR1*, *ZRT2*, and *CTR1*, genes which encode iron, zinc, and copper transporters [41]. Either alone or in combination with itraconazole, aprepitant increased intracellular ROS levels, impairing ROS detoxification systems likely through its interference with metal ion transport [41]. Interestingly, iron, but not copper, supplementation rescued the growth of *C*. *auris* cells treated with the drug combination, suggesting iron homeostasis as a key mechanism for the synergistic effect. This drug combination also showed efficacy against other *Candida* species and rescued host survival in a *C*. *elegans* model of fungal infection [41]. Screening of the Pharmakon library identified ospemifene, an oestrogen receptor modulator, which interfered with ABC (ATP-binding cassette) and MFS (major facilitator superfamily)-mediated efflux [42]. In combination with itraconazole, ospemifene decreased *C*. *albicans* and *C*. *auris* fungal burdens in *C*. *elegans* infections [42]. However, the potential deployment of ospemifene as an azole potentiator is unclear given its reported adverse effects [42]. In a similar manner, proton pump inhibitors (PPIs), such as omeprazole and rabeprazole, inhibited ABC-mediated efflux and increased the survival of *G*. *mellonella* in a *C*. *albicans* infection [43]. They also blocked filamentation, biofilm formation and inhibited *C*. *albicans* phospholipase activity [43]. Due to their increased safety profile, PPIs could represent an exciting development in antifungal potentiators.

Interestingly, statins, used to control cholesterol levels, have emerged as promising azole potentiators by disrupting the mevalonate pathway in sterol biosynthesis [44]. One statin, pitavastatin, also interfered with ABC efflux pumps [45]. While several statins reduced fungal burdens in animal models of *Candida* infection, the effects of atorvastatin were unconclusive [44]. The use of statins in human clinical trials was associated with lower mortality, while others reported no impact on outcomes, indicating that additional controlled studies are required to establish their efficacy in human infections [44].

Several neuroactive compounds can also potentiate azoles, although most studies focused on demonstrating their in vitro and in vivo synergistic effects, with little mechanistic insight, as in the case of sertraline or bromperidol [46,47]. Derivatives of haloperidol, an antipsychotic, synergized with fluconazole and inhibited filamentation, biofilm formation, as well as increased survival of mice infected with drug-resistant *C*. *albicans* [48]. The drug combination decreased *ERG11* expression and altered sterol composition, impacting membrane integrity, but also down-regulated *MDR1* expression, decreasing drug efflux [48]. Fluoxetine, an SSRI (selective serotonin reuptake inhibitor), decreased the activity of phospholipases and secreted aspartyl proteases (*SAP1-4*) in *C*. *albicans* [49]. The fluoxetine/fluconazole combination interfered with biofilm formation, decreased fungal burdens, and increased *G*. *mellonella* survival during *C*. *albicans* infection [49]. Dicyclomine, an anticholinergic drug, potentiated fluconazole against *C*. *auris*, presumably by inhibiting Golgi trafficking, disrupting ergosterol synthesis and nutrient transport [46]. Finally, clorgyline and its analogues also synergized with azoles in several *Candida* species and reduced drug efflux by inhibiting ABC and MSF transporters [50,51]. However, the on-target effects of neuroactive compounds could outweigh their benefits as potentiators, highlighting the challenges faced by repurposed drugs during development. Nevertheless, exploring approved molecules can uncover novel synergy routes and can provide initial scaffolds for developing antifungal potentiators.

## Novel compounds

Multiple recent efforts have also been directed towards the screening of diverse compound collections. For example, azoffluxin was discovered by screening the library of the Boston University’s Centre for Molecular Discovery [52]. Azoffluxin potentiated fluconazole against most *C*. *auris* isolates but did not affect those with *ERG11* mutations or those which overexpressed *MDR1* [52]. The combination was also active on fluconazole-resistant *C*. *albicans* strains [52]. In both species, the compound potentiated fluconazole by inhibiting the efflux pump Cdr1 and increasing intracellular drug accumulation [52]. Assays on human cell lines indicated minimal cytotoxicity, while experiments on mice showed good tolerability and decreased *C*. *auris* fungal burdens [52]. Overall, azoffluxin appears to be a promising candidate for further development.

Another screen revealed 1,4-benzodiazepines as azole potentiators, a set of small molecules without intrinsic antifungal activity [53]. Here, the drug combinations synergized with azoles, inhibited filamentation, and improved the survival of *C*. *albicans*-infected *G*. *mellonella* [53]. As the compounds potentiated other inhibitors of ergosterol and sphingolipid biosynthesis, their activity was ascribed to the perturbation of lipid homeostasis [53]. Interestingly, when tested against other *Candida* species, the compounds were more effective in species closely related to *C*. *albicans* (e.g., *Candida tropicalis* and *Candida dublinensis*) relative to phylogenetically distant species such as *C*. *auris* or *N*. *glabrata* [53]. While the 1,4-benzodiazepine potentiators were not cytotoxic to mammalian cells, their species-specific effects warrant further development and optimization.

Another study developed II-6s, a small antimicrobial derivative with potent antifungal activity against *C*. *albicans*, which also synergized with azoles [54]. The combination therapy reduced drug efflux, inhibited filamentation, and was fungicidal [54]. Its activity was ascribed to mitochondrial damage and the down-regulation of the Hog1 MAPK pathway, but no direct target was identified [54]. However, II-6s had a relatively high cytotoxicity [54], indicating that additional optimization would be needed prior to in vivo experiments.

New chemical targets interfering with azole resistance can also be discovered through genetic screening. One *S*. *cerevisiae* screen identified several vesicular transport genes impacting ergosterol levels and fluconazole susceptibility, prompting the question of whether inhibiting this process could potentiate azoles [55]. To test this hypothesis, the authors used sortin2 and sortin3, which inhibit endosomal trafficking in *S*. *cerevisiae* [56]. Interestingly, sortin3 antagonized fluconazole in *C*. *albicans*, but sortin2 synergized with fluconazole in both *C*. *albicans* and *N*. *glabrata* [55]. While these results are encouraging, sortin2 requires the identification of its precise molecular target in *Candida* species. Due to the lack of testing on mammalian models, it is challenging to anticipate its toxicity since it may also target conserved elements of mammalian vesicular transport.

Active compounds were also identified through protein–protein interaction screens, as in the case of iKIX1, a molecule that selectively blocks the interaction between the *N*. *glabrata* Pdr1 activation domain and the Mediator complex, thereby blocking the expression of Pdr1-regulated genes [57]. In *N*. *glabrata*, Pdr1 is a key regulator of drug resistance genes, including efflux pumps [58]. Yeast cells treated with iKIX1 regained azole susceptibility, both in vitro and in vivo, suggesting that azole resistance can be tackled by interfering with Pdr1 activity [57]. Although the compound appeared inert in a murine model [57], toxicity studies are necessary to evaluate the full potential of this molecule. A chemo-structural approach yielded CMLD013075, a highly fungal-selective inhibitor of Hsp90 [59], one of the key modulators of azole resistance [10]. This molecule synergized with fluconazole rendering it cidal [59]. While CMLD013075 was not toxic to mammalian cells, it was challenging to dose [59], suggesting that additional pharmacological optimization studies would be necessary.

Consequently, multiple compounds described above require extensive development to attain PK/PD properties suitable for clinical trials. Animal toxicity and adverse drug–drug interactions might also arise with novel compounds. Nonetheless, exploring diverse compound libraries for their ability to combat *Candida* species can uncover molecules with distinct modes of action, broad-spectrum activity, or fungicidal properties.

## The future of azole combinations

The guidelines for treating candidiasis do not currently include combination therapies with azole drugs. However, such combinations could represent a game-changer for managing recalcitrant *Candida* infections. Moreover, the discovery of new molecules with antifungal properties can expand our understanding of fungal cell biology. Indeed, the compounds highlighted here target diverse processes, including drug efflux, lipid homeostasis, vesicular trafficking, metabolism, mitochondrial function, and cell wall or membrane integrity (Fig 1 and Table 1). The disruption of these processes could be leveraged for synergy with azole activity. However, additional mechanistic, PK/PD and clinical efficacy data will be essential to establish whether azole combinations can become a reliable treatment option for *Candida* infections.

**Table 1:** Examples of azole potentiators, the *Candida* species where these are active, and their identified or proposed modes of action.

Potentiator name	Species in which the potentiator is active	Mechanism of action	Intrinsic antifungal activity	Synergy with other drugs	Type of activity	Animal model tested	References
**Natural products**							
beauvericin	*C*. *albicans*, *N*. *glabrata*, *C*. *tropicalis*, *C*. *krusei*	inhibition of drug efflux, inhibition of Hsp90 activity	no	-	cidal	*M*. *musculus*	[9,11]
catechol	*C*. *albicans*	increased farnesol production, inhibition of the Ras1 pathway	yes	polyenes	static	-	[25,26]
copper	*C*. *albicans*, *N*. *glabrata*	decreased efflux, inhibition of ergosterol synthesis	no	-	static	-	[29–31]
cyclosporine	*C*. *albicans*	inhibition of calcineurin pathway, reduced expression of drug resistance genes	no	-	cidal	-	[14–16]
CZ66	*C*. *albicans*, *N*. *glabrata*, *C*. *parapsilosis*, *C*. *tropicalis*, *C*. *guilliermondii*	inhibition of alternative ergosterol pathway via Erg251	no	-	cidal	-	[27]
milbemycin derivatives	*C*. *albicans*, *N*. *glabrata*	inhibition of drug efflux, increased ROS production	yes	-	cidal	*M*. *musculus*	[18]
propolis	*C*. *albicans*, *N*. *glabrata*, *C*. *paraspilosis*, *C*. *krusei*	disruption of the cell wall and membrane integrity, impairment of mitochondrial function	no	-	static	-	[22,23]
NPD827	*C*. *albicans*, *C*. *auris*	disruption of membrane integrity	no	ergosterol biosynthesis inhibitors	static	*C*. *elegans*	[21]
turbinmicin	*C*. *auris*, *C*. *albicans*, *C*. *tropicalis*, *N*. *glabrata*	disruption of vesicular trafficking	yes	-	cidal	*M*. *musculus*	[19,20]
**Repurposed drugs**							
aprepitant	*C*. *auris*, *C*. *albicans*, *C*. *krusei*, *C*. *parapsilosis*, *C*. *tropicalis*	disruption of metal homeostasis, down-regulation of glucose transporters, impairment of ROS detoxification	no	-	cidal	*C*. *elegans*	[41]
atazanavir, darunavir	*C*. *albicans*, *C*. *auris*	down-regulation of *SAP2* and *BCR1*, inhibition of filamentation, inhibition of drug efflux	yes	-	static	*G*. *mellonella*, *M*. *musculus*	[39,41]
clorgyline derivatives	*C*. *albicans*, *C*.*auris*, *N*. *glabrata*	inhibition of drug efflux	no	-	cidal	-	[50,51]
dicyclomine	*C*. *auris*	inhibition of Golgi trafficking, disruption of ergosterol synthesis and of nutrient transport	unknown	-	unknown	-	[46]
fluoxetine	*C*. *albicans*, *C*. *krusei*, *N*. *glabrata*, *C*. *tropicalis*, *C*. *parapsilosis*	inhibition of phospholipase and protease activity	no	-	static	*G*. *mellonella*	[49]
haloperidol derivatives	*C*. *albicans*, *N*. *glabrata*	decreased drug efflux, decreased *ERG11* expression, altered sterol composition	yes	-	static	*M*. *musculus*	[48]
lopinavir	*C*. *auris*, *C*. *albicans*, *N*. *glabrata*, *C*. *krusei*, *C*. *parapsilosis*, *C*. *tropicalis*	down-regulation of glucose transporters, lowering of ATP, inhibition of drug efflux	yes	-	cidal	*C*. *elegans*	[38]
ospemifene	*C*. *auris*, *C*. *albicans*, *N*. *glabrata*	inhibition of drug efflux	no	-	static	*C*. *elegans*	[42]
pitavastatin	*C*. *auris*, *C*. *albicans*, *N*. *glabrata*	inhibition of drug efflux	no	-	cidal	*C*. *elegans*	[45]
proton pump inhibitors	*C*. *albicans*	inhibition of drug efflux and of phospholipase activity	no	-	unknown	*G*. *mellonella*	[43]
statins	*C*. *albicans*, *C*. *tropicalis*, *N*. *glabrata*, *C*. *krusei*, *C*. *parapsilosis*, *C*. *utilis*	inhibition of mevalonate pathway in ergosterol biosynthesis	some compounds	polyenes, echinocandins	static	*M*. *musculus*, *O*. *cuniculus*	[44]
sulfonamides	*C*. *albicans*, *C*. *auris*	inhibition of folate synthesis	no	-	static	*C*. *elegans*	[35,36]
**Novel compounds**							
1,4-benzodiazepines	*C*. *albicans*, *C*. *parapsilosis*, *C*. *tropicalis*, *C*. *orthopsilosis*, *C*. *metapsilosis*, *C*. *dublinensis*	disruption of lipid homeostasis	no	-	static or cidal	*G*. *mellonella*	[53]
azoffluxin	*C*. *auris*, *C*. *albicans*	inhibition of *CDR1* efflux pump	no	intracellular antifungals	cidal	*M*. *musculus*	[52]
CMLD013075	*C*. *albicans*	inhibition of Hsp90 activity	no	-	cidal	*M*. *musculus*	[59]
iKIX1	*N*. *glabrata*	inhibition of Pdr1 activity	no	-	cidal	*G*. *mellonella*, *M*. *musculus*	[57]
II-6s	*C*. *albicans*	inhibition of drug efflux and Hog1 pathway, mitochondrial damage	yes	-	cidal	-	[54]
sortin2	*C*. *albicans*, *N*. *glabrata*	inhibition of vesicular trafficking	no	-	static	-	[55]

Information on their intrinsic antifungal activity, their ability to synergize with other antifungals, the type of activity elicited in combination with azoles, and whether the molecules were tested in animal models of infection is also included.
